# Microbial-Based Bioactive Compounds to Alleviate Inflammation in Obesity

**DOI:** 10.3390/cimb46030119

**Published:** 2024-02-28

**Authors:** Oladayo Emmanuel Apalowo, Grace Adeola Adegoye, Tolulope Mobolaji Obuotor

**Affiliations:** 1 Department of Food Science, Nutrition and Health Promotion, Mississippi State University, Starkville, MS 39762, USA; oea24@msstate.edu; 2 Department of Nutrition and Health Science, Ball State University, Muncie, IN 47306, USA; 3 Department of Microbiology, Federal University of Agriculture, Abeokuta 110001, Nigeria; obuotortm@funaab.edu.ng

**Keywords:** obesity, inflammation, functional foods, adipose tissue, metabolic disease, probiotics, prebiotics, synbiotics, postbiotics

## Abstract

The increased prevalence of obesity with several other metabolic disorders, including diabetes and non-alcoholic fatty liver disease, has reached global pandemic proportions. Lifestyle changes may result in a persistent positive energy balance, hastening the onset of these age-related disorders and consequently leading to a diminished lifespan. Although suggestions have been raised on the possible link between obesity and the gut microbiota, progress has been hampered due to the extensive diversity and complexities of the gut microbiota. Being recognized as a potential biomarker owing to its pivotal role in metabolic activities, the dysregulation of the gut microbiota can give rise to a persistent low-grade inflammatory state associated with chronic diseases during aging. This chronic inflammatory state, also known as inflammaging, induced by the chronic activation of the innate immune system via the macrophage, is controlled by the gut microbiota, which links nutrition, metabolism, and the innate immune response. Here, we present the functional roles of prebiotics, probiotics, synbiotics, and postbiotics as bioactive compounds by underscoring their putative contributions to (1) the reduction in gut hyperpermeability due to lipopolysaccharide (LPS) inactivation, (2) increased intestinal barrier function as a consequence of the upregulation of tight junction proteins, and (3) inhibition of proinflammatory pathways, overall leading to the alleviation of chronic inflammation in the management of obesity.

## 1. Introduction

The issue of obesity is multifaceted and extremely complex, as it increases the prevalence of type 2 diabetes mellitus (T2DM), cardiovascular diseases, non-alcoholic fatty liver disease, and related diseases, and has now emerged as a serious worldwide health concern [1,2]. According to the World Obesity Atlas, in America, obesity prevalence among children and adolescents is likely to rise from 2020 to 2035, notably among boys, where the incidence is forecast to rise from 20% to 33%. Both men and women are likely to experience an increase in the prevalence of obesity over the same 15-year period, with almost half of all adults (47% to 49%) predicted to be affected by the disease by 2035 [3]. Globally, more than 4 billion people are projected to be affected by 2035 (BMI ≥ 25 kg/m^2^), reflecting over 50% of the world’s population compared to 2.6 billion in 2020, with the prevalence of obesity anticipated to rise from 14% to 24% in the population during the same period. Environmental variables, genetics, and energy imbalance—a state in which energy intake exceeds energy expenditure—can all have an impact on body weight [1]. The aging process is correlated with an augmentation in abdominal white adipose tissue (WAT) and the deposition of fat in skeletal muscle, significantly impacting insulin sensitivity [4,5]. Alterations in the lifestyle of the elderly, particularly as they transition into retirement, may induce a chronic positive energy balance, resulting in excess fat tissue accumulation. This condition accelerates the onset of age-related diseases [6]. It is increasingly evident that an obese state is associated with a diminished lifespan and health consequences akin to those observed in advanced aging [7]. Additionally, several studies have linked chronic inflammation to obesity and obesity-associated diseases [8,9,10].

The gut microbiota, consisting of approximately 100 trillion bacteria, colonizes the human intestine and plays a pivotal role in metabolic processes by producing many enzymes essential for extracting energy from the host’s diet and facilitating energy deposition in fat stores [11,12,13]. This functionality is contingent upon maintaining a delicate equilibrium between potentially pathogenic bacteria and many nonpathogenic microorganisms that contribute to overall health [14]. The commensal bacteria inhabiting the gut offer the advantages of functioning as an auxiliary organ, actively participating in cellulose digestion, and enhancing the development and maturation of both the intestinal and systemic immune systems [15]. The host’s immune system and metabolic pathways can be influenced by the gut microbiota in response to food preferences and energy requirements because of its flexibility and adaptability [16]. This dynamic relationship is essential to immunity and metabolism and has a major influence on optimal health. Age-related alterations may lead to systemic inflammaging, which might affect the makeup of the gut microbiota due to its continuous contact with organs and tissues, which, in turn, alters intestinal immune responses [17,18]. Research indicates variations in the composition of intestinal flora between lean and overweight rodents and humans [19,20,21], while various datasets from diverse sources have substantiated a causative association between gut microbiota and obesity [12,19,22,23].

Probiotics, prebiotics, synbiotics, and postbiotics, such as short-chain fatty acids (SCFAs) and muramyl dipeptide, have been shown to have a significant impact on metabolic function [24]. Nonetheless, there is a significant study gap on the functional involvement of these components in the alleviation of chronic inflammation in the context of obesity management. Hence, this review explores the functional roles of prebiotics, probiotics, synbiotics, and postbiotics, with a focus on their overall effects on gut microbiota, functioning as bioactive compounds in the alleviation of chronic inflammation in obesity and related diseases.

## 2. Gut Microbiota, Inflammation, and Obesity

### 2.1. The Gut Microbiota

The human gut microbiota, a complex ecosystem consisting of up to 100 trillion microorganisms, engages in a largely symbiotic relationship with its host [25]. This diverse microbial community, collectively termed the microbiome, harbors at least 150 times more genes than the human genome itself [25]. Analyses based on 16S rRNA-targeted sequencing reveal that fecal samples from healthy individuals predominantly harbor two major bacterial phyla, namely the Gram-negative Bacteroidetes consisting of the genera *Bacteroides*, *Prevotella*, *Parabacteroides*, and *Alistipes* and the Gram-positive Firmicutes made up of the *Faecalibacterium prausnitzii*, *Eubacterium rectale*, and *Eubacterium hallii* species in addition to numerous low-abundance species [26,27].

Notably, the gut microbiota exhibit remarkable microbial and genetic diversity, with specific bacterial species associated with distinct gastrointestinal regions. Bacterial density steadily increases from the proximal to the distal portions of the gastrointestinal tract [27]. The stomach harbors the lowest abundance, with approximately 101 microbial cells per gram of content. This number rises to 103 in the duodenum, 104 in the jejunum, and 107 in the ileum and culminates at 1012 cells per gram in the colon [28]. Consequently, the large intestine houses over 70% of the body’s microorganisms, which have a symbiotic relationship with the host and exert a substantial influence on the overall well-being of the host [29].

Healthy aging comprises limiting age-related health problems in older people by avoiding or delaying chronic diseases, even though the chance of various ailments grows with age due to the loss of tissue structure and physiological function [30,31]. This underscores the critical role that these variables play in age-related morbidity [30]. Aging and health status affect biological parameters, with the human microbiota being a dynamic indication that is modified by factors such as nutrition, lifestyle, and immunological response [32]. The varying composition of the gut microbiota in older adults reflects their current and previous health situations [30].

The gut microbiota is essential for development and adult homeostasis, and alterations have been associated with inflammatory and metabolic problems in adults, including inflammatory bowel disease and obesity [20,33,34,35]. While the gut microbiota remains stable and individual-specific in healthy adults, there is a significant fluctuation in older individuals compared to younger people [36,37]. Immunosenescence, which is characterized by persistent NF-kB-mediated inflammation and the loss of naïve CD41 T cells, is one facet of the aging process [38]. Intestinal homeostasis is significantly influenced by gut microbiota, and immunosenescence is linked to the persistent activation of the innate and adaptive immune systems [39,40].

Commensal bacteria control an innate immune response and accelerate the host’s response to enteric infections [41] by increasing baseline pro-inflammatory IL-1β production, which exerts a protective effect in assisting gut pathogen clearance and neutrophil recruitment by stimulating the expression of endothelial adhesion molecules [42]. Additionally, natural Killer (NK) T cells are regulated by commensal bacteria that can express both T cell receptors and NK cell receptors, which help to maintain homeostasis, and promote the release of inflammatory cytokines, including TNF-α, IFN-γ, IL-2, IL-4, IL-13, IL-17A, IL-21, and inhibit excessive inflammatory response [43,44].

Correlations between certain microbiota elements and an increase in pro-inflammatory cytokines, such as serum TNF-α, IL-6, IL-8, and the C-reactive protein (CRP), have thus demonstrated this relationship in the elderly [35]. Additionally, mitogens and LPSs cause macrophages to secrete more IL-6 and IL-8, which alters macrophage activity [30]. Age-related dysregulation brought on by gut microbiota dysbiosis may weaken the intestinal barrier and cause the release of microbial products that raise pro-inflammatory factors like TNF-α, interferons, IL-6, and IL-1 [45]. This, in turn, may contribute to a chronic low-grade inflammatory state linked to chronic diseases [18,46,47,48].

### 2.2. Obesity and Related Diseases

Obesity, defined as an abnormal or excessive accumulation of fat, has reached global pandemic proportions [49]. The current clinical practice for identifying overweight and obese individuals relies primarily on body mass index (BMI) [50]. Accordingly, based on established BMI classifications, values between 25 and 29.9 kg/m^2^ classify individuals as overweight, while a value exceeding 30 kg/m^2^ indicates obesity [51]. Obesity primarily arises from an energy imbalance, where caloric intake exceeds expenditure, which leads to the storage of excess energy, such as fat and glycogen, in subcutaneous adipose tissue (SAT) and organs [52,53]. However, adipose tissue itself exhibits functional heterogeneity, comprising distinct depots with specialized roles [54].

Adipose tissue is classified into WAT and brown adipose tissue (BAT), distinguished by variations in morphology, anatomical position, developmental patterns, and metabolic functions [55]. WAT serves as a key endocrine organ, storing energy in the form of triglycerides and secretes adipokines, while BAT, characterized by multilocular adipocytes and UCP-1 expression, actively contributes to energy expenditure through non-shivering thermogenesis, playing a role in regulating body temperature and providing protection against obesity [56]. WAT is categorized into two primary depots, visceral WAT (VAT) and SAT, both of which are extensively examined for their correlation with the development of related diseases [57]. Despite accounting for just 1% to 2% of total fat, BAT is indispensable for maintaining homeostasis and has a beneficial impact on blood glucose levels [58]. Obese and diabetic individuals have smaller BATs and less activity than those with a normal BMI [59].

Adipose tissue in individuals exhibiting overweight or obesity is intricately associated with a chronic, low-grade inflammatory state, characterized by the heightened infiltration of macrophages of the M1 or ‘classically activated’ phenotype from the circulation into adipose tissue, leading to adipose tissue inflammation through the release of pro-inflammatory cytokines (TNF-α, IL-6, IL-8), while the balance of anti-inflammatory cytokines (IL-4, IL-10, IL-13, IL-19) from adipocytes tends to decrease with weight gain, favoring the heightened production of pro-inflammatory adipokines [60,61,62]. Adipose tissue not only releases adipokines (leptin, adiponectin, visfatin, resistin) and constituents of the extracellular matrix to modulate interconnected pathways but also undergoes hyperplasia and hypertrophy due to excess fat accumulation, altering the secretome, releasing metabolites, and subsequently influencing the surrounding microenvironment [63,64].

An elevated level of proinflammatory adipokine leptin, in conjunction with an increase in the levels of the hepatocyte growth factor (HGF), plasminogen activator inhibitor-1 (PAI-1), resistin, TNF-α, IL-1β, IL-6, and monocyte chemoattractant protein-1 (MCP-1), accompanied by a simultaneous decrease in adiponectin, contribute to the metabolic syndrome, which is characterized by glucose intolerance, insulin resistance, central obesity, dyslipidemia, hypertension, heightened cardiovascular disease risk, and increased susceptibility to cancer [8,9,10]. Increased serum levels of free fatty acids (FFAs) in obese individuals promote vascular endothelial growth factor A (VEGF-A) and vimentin expression through peroxisome proliferator-activated receptor gamma (PPARγ) upregulation, contributing to tumor growth, insulin resistance, and hepatic steatosis. At the same time, the concomitant overexpression of TNF-α and leptin inhibits insulin receptor activation, inducing resistance in the muscle, liver, islet α-cells, and adipose tissue, leading to T2DM [65,66].

### 2.3. Low-Grade Chronic Inflammation: Linking Gut Microbiota and Obesity

Obesity is characterized by changes in the abundant ratios of the dominating phyla. While some research implies that obese individuals have a higher Firmicutes to Bacteroidetes ratio, the consistency of this observation and its reliability as a biomarker remains uncertain [67,68,69]. Furthermore, obesity-linked low-grade inflammatory states may be aggravated by microbiota-associated inflammatory processes [69]. Seven aging pillars that form an interconnected network that converge at inflammation have been identified [70], with dysfunction in one pillar leading to inflammation and subsequently impacting other pillars [18]; this event is now referred to as inflammaging, defined as a “chronic, sterile (occurring in the absence of infection and primarily driven by endogenous signals), low-grade inflammation that occurs during aging” [18]. This chronic inflammatory state, characterized by the innate immune system via macrophage activation and regulated by the gut microbiota, results in the production of inflammatory products [18,71].

Previous research demonstrated that a 4-week high-fat (HF) diet resulted in a two-to-threefold increase in plasma LPS levels, which is comparable to the effects observed during the subcutaneous infusion of LPSs in mice, leading to insulin resistance and obesity [72]. LPSs, a powerful activator of Toll-like receptor 4 (TLR4), are found in Gram-negative bacteria [73]. Hence, changes in gut microbiota composition, known as intestinal dysbiosis, may contribute to a persistent low-grade inflammatory response in obesity. Since LPSs contain lipid A, they can translocate across the intestinal mucosa via tight junctions or with chylomicron facilitation. Given that lipoproteins play a crucial role in the absorption and transport of dietary triglycerides, this mechanism may serve as an initiating factor for inflammation, potentially contributing to the commonly observed insulin resistance in obesity [72,74].

**Figure 1:**
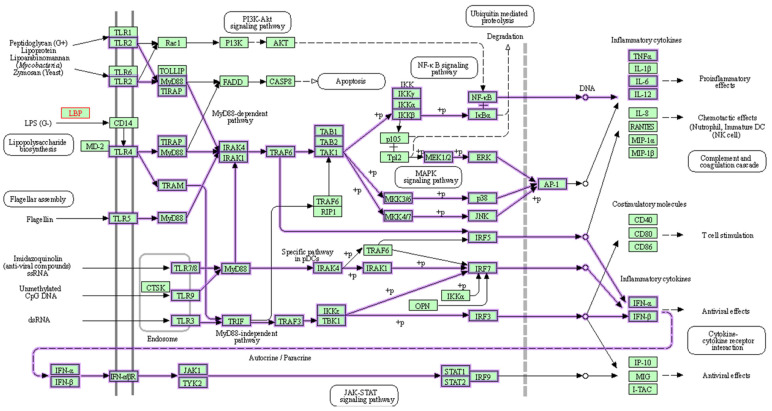
LPSs activate TLR4, signaling a chain reaction that releases inflammatory cytokines. TLRs, which are homologs of Toll in Drosophila, are present on the membranes of innate immune cells in humans (e.g., macrophages and dendritic cells) and are activated by membrane components from Gram-positive or Gram-negative bacteria. When TLRs detect pathogens, they immediately activate innate immunity, causing the generation of proinflammatory cytokines and increasing the expression of costimulatory molecules. As shown above, TLR signaling networks include a MyD88-dependent pathway that rapidly activates NF-kB and MAPK, resulting in the generation of proinflammatory cytokines, while the MyD88-independent pathway is linked to delayed NF-kB and MAPK activation, resulting in the stimulation of IFN-beta, IFN-inducible genes, and dendritic cell maturation. Green box represents organism-specific pathways; +p = phosphorylation [76].

As a member of the TLR family, TLR4 is found in many different types of cells, including macrophages. It recognizes pathogen-associated molecular patterns (PAMPs) and initiates a complicated cell signaling pathway that, when bound by LPSs, activates inflammatory response, and triggers the release of cytokines provided by the KEGG pathway in Figure 1 [13,75,76]. Additionally, TLR4 has been linked to the inflammatory response associated with increased intestinal permeability in circumstances such as diet-induced obesity (DIO), which leads to insulin resistance and metabolic imbalance [13]. Furthermore, elevated LPS levels are associated with increased intestinal permeability, driven by the reduced expression of vital tight junction proteins like zonula occludens-1 (ZO-1), claudin, and occludin, leading to a compromised epithelial barrier that facilitates the entry of bacterial components from the intestinal lumen into the circulation, potentially initiating inflammation and insulin resistance in humans and animals [77,78].

### 2.4. Short-Chain Fatty Acids (SCFAs)

The fermentation of non-digestible carbohydrates in the cecum and colon by the gut microbiota produces SCFAs, such as acetate, propionate, and butyrate, demonstrating metabolic cooperation among the bacterial community, where the collective role of the entire community is emphasized, and absorbed SCFAs in the intestine occur via passive diffusion via monocarboxylate transporter 1 (MCT1) [79]. SCFA, particularly butyrate, is a primary source of energy for colonic epithelial cells, promoting cell proliferation and differentiation [80,81], whereas acetate and propionate play separate roles in cholesterol/fatty acid precursor and gluconeogenesis, respectively [82]. While other bacterial by-products, such as conjugated linoleic acids and bile acids, and gases, including methane and hydrogen sulfide, have metabolic regulatory activities, they play limited roles in mammalian physiology in comparison to SCFA’s dominant effect [83,84].

Butyrate and acetate are essential for maintaining epithelial barrier function by influencing tight-junction protein expression (zonulin and occludin), increasing mucus production, and reducing intestinal permeability, with acetate having the most pronounced effects on epithelial protection and both SCFAs contributing to increased fatty acid oxidation and energy expenditure, potentially leading to weight loss, insulin sensitivity, and improved metabolic health [85,86]. SCFAs block NF-kB activation in host immune cells via binding to the G-protein-coupled receptors 43 and 41 (GPR43 and GPR41), with GPR43 playing an important role in regulating the anti-inflammatory responses elicited by acetate [87,88].

## 3. Probiotics, Prebiotics, Synbiotics, and Postbiotics in the Management of Obesity and Related Diseases

### 3.1. Probiotics

Probiotics are live microorganisms that, when ingested in appropriate amounts, confer a health benefit [89]. Their decline has been linked to an elevated risk of immune–metabolic conditions such as obesity, T2DM, and metabolic syndrome [89,90,91]. The two most frequent genera are Lactobacillus and Bifidobacterium [92]. Despite the absence of approval from medical regulatory authorities like the European Food Safety Authority and the US Food and Drug Administration for any probiotic formulation as a therapeutic agent [93,94,95], in compliance with the guidelines established by the Ministry of Food and Drug Safety (MFDS) for healthful functional foods, South Korea uses 19 probiotic species as functional ingredients. Of these, 4 species are Bifidobacteria, and 12 are members of the Lactobacilli genus [96]. Certain microbial species like *Akkermansia muciniphila*, *Faecalibacterium prausnitzii*, *Anaerobutyricum hallii* and *Anaerobutyricum soenhgenii*, *Bacteroides uniformis*, *Bacteroides coprocola*, *Parabacteroides distasonis*, *Parabacteroides goldsteinii*, *Hafnia alvei*, *Odoribacter laneus*, and *Christensenella minuta* have been identified as potential next-generation probiotics or live biotherapeutic products [90,97,98,99]. These strains hold promise, particularly in addressing obesity and related disorders, with some being prevalent in the microbiota of most individuals.

**Figure 2:**
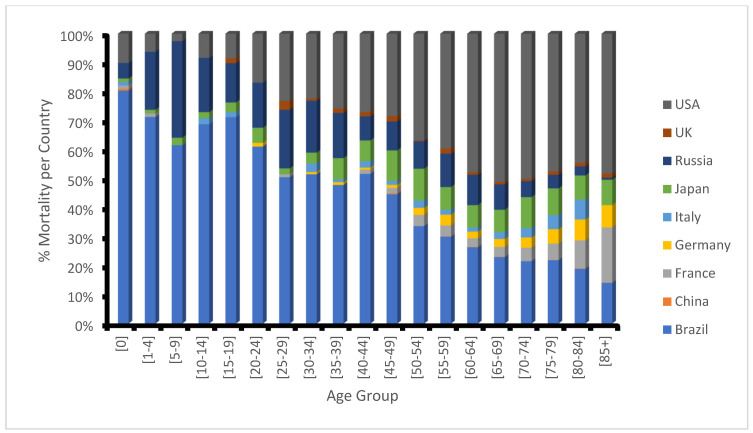
Microbial dysbiosis may lower the intake of nutrients and increase total mortality due to nutritional deficiencies. This causes protein–energy malnutrition, iodine deficiency, vitamin A deficiency, iron deficiency anemia, and other nutritional disorders. Inadequate consumption/availability of essential macronutrients or micronutrients can result in fatal outcomes. The latest data included by country: China, France—2017; Italy, Russia—2019; Brazil, Germany, Japan, UK, USA—2020 [107,108].

Despite increased dietary intake, the global rise in obesity and diabetes is associated with prevalent micronutrient deficiencies among obese individuals, specifically in vitamins and minerals important for glucose metabolism and insulin signaling pathways, potentially contributing to the development of diabetes and fatal outcomes (Figure 2) [100]. The small intestine, which consists of the duodenum, jejunum, and ileum, is the primary location for macro- and micronutrient digestion and absorption. A bidirectional relationship between the gut microbiome and micronutrients involves microbial reliance on micronutrients for growth and metabolism while also producing essential vitamins such as vitamins B and K, facilitating mineral absorption. Although microbial dysbiosis may influence nutrient bioavailability, probiotic supplements, including lactic acid bacteria and Bifidobacterium, have been shown to promote beneficial microbial populations, enhance barrier integrity, and alleviate nutrient malabsorption and small intestinal disease [101,102,103,104,105,106].

Their impact on various physiological markers has been identified. For example, the Lactobacillus species administered to diet-induced obese mice resulted in beneficial outcomes, including reduced weight, visceral fat, glucose, insulin, triglyceride levels, insulin resistance, and proinflammatory cytokines, accompanied by increased IL-10 and improved fatty liver indices [109,110,111]. In overweight or grade 1 obese adults, a 12-week *L. gasseri* supplementation led to significant reductions in visceral fat and waist circumference [112], while a 24-week L. rhamnosus treatment resulted in significant weight reductions in female participants with obesity [113]. The oral administration of Bifidobacterium longum NK49, Lactobacillus plantarum NK3, and Bifidobacterium longum PI10 improved obesity in mice by improving intestinal barrier integrity via glucagon-like peptide 1 (GLP1) and IL-10 induction, modulating immune cells, and lowering TNF-α expression [114,115].

Another recent randomized controlled trial on 50 obese women (mean age: 55.2 ± 6.9 years; BMI: 36.6 ± 6.0 kg/m^2^) showed that the administration of a probiotics supplement for 12 weeks consisting of *Bifidobacterium bifidum* W23, *Bifidobacterium lactis* W51, *Bifidobacterium lactis* W52, *Lactobacillus acidophilus* W37, *Lactobacillus brevis* W63, *Lactobacillus casei* W56, *Lactobacillus salivarius* W24, *Lactococcus lactis* W19, and *Lactococcus lactis* W58 improved the lipid profile, and significantly reduced homocysteine, TNF-α, total cholesterol, LDL-c, and triglyceride with an increase in total antioxidant status. However, no significant change in BMI, waist circumference, SBP, or DBP was observed [116]. Similarly, an earlier study involving 81 obese women (mean age 55.16 ± 6.87 years; BMI: 36.57 ± 5.95 kg/m^2^) reported that a high dose of the probiotic supplement for 12 weeks resulted in a decrease in BMI, systolic blood pressure (SBP), diastolic blood pressure (DBP), VEGF, IL-6, TNF-α, thrombomodulin, pulse wave analysis systolic pressure, pulse wave analysis pulse pressure, pulse wave analysis augmentation index, and pulse wave velocity [117].

A study on 58 obese postmenopausal women (mean age: 61.4 ± 6 years; BMI: 34.2 ± 3.1 kg/m^2^) with the administration of the probiotic supplement *L. paracasei* F19 for 6 weeks showed alterations in the fecal abundance of two metagenomic species (*Eubacterium rectale* and *Ruminococcus torques*). However, no significant effect was observed for insulin sensitivity, lipid metabolism, inflammatory markers, or anthropometric measures [118]. Likewise, probiotics *Lactobacillus acidophilus* La5 and *Bifidobacterium animalis* subsp lactis Bb12 administered to 156 overweight men and women (mean age: 68.4 ± 8 years; BMI: 31 ± 4 kg/m^2^) for 6 weeks showed no significant change in anthropometric measures, insulin, or HbA1c, and no improvement in glycemic control [119].

To date, investigations derived from animal models (Table 1) and clinical trials (Table 2) have underscored a prevalent trend wherein the amelioration of inflammatory indicators emerges as a notable feature linked to the favorable actions of probiotics in rectifying metabolic dysregulations associated with obesity and related diseases.

### 3.2. Prebiotics

Prebiotics are described as non-digestible dietary components that selectively promote the growth and activity of specific beneficial bacteria in the colon that boost human health [160]. As defined by four criteria in 2004, prebiotics resist digestion by mammalian enzymes, solely undergo fermentation by the gut microbiota, elicit beneficial effects either systemically or within the luminal environment, and selectively promote the growth of gut microbiota linked to optimal health [160,161]. Various natural sources and suggested substances, such as galacto-oligosaccharides and inulin-type fructans, act as prebiotics, positively influencing gut microbiota composition and health outcomes, with evidence indicating that prebiotic-rich diets are associated with lower food intake, reduced body fat composition and weight gain, especially in overweight and obese individuals [86,162]. Prebiotics such as oligofructose stimulate the production of SCFAs and increase the number of enteroendocrine cells (EECs), resulting in the release of peptides vital to lipid elimination [163].

Furthermore, prebiotics impact the gut microbiota, resulting in a lower presence of LPSs and improving the structural integrity of the intestinal barrier. This fortification functions as a prophylactic strategy, preventing bacterial translocation into the circulation and causing systemic inflammation [164,165]. Prebiotics confer several health benefits impacting lipid and glucose metabolism, intestinal microbiota composition, obesity, and satiety hormones [163] in addition to immunological regulation, which is characterized by increased levels of immune-regulatory interleukins and intestinal-specific immunoglobulins, as well as a decrease in pro-inflammatory interleukins [166,167]. In addition, acorn and sago polysaccharides and unsaturated alginate oligosaccharides demonstrate an ability to reduce mucosal inflammatory biomarkers and alleviate gut hyperpermeability in obese and type 2 diabetic mice. They improve the intestinal barrier in obese mice by increasing ZO-1 and occludin expressions, respectively [168].

### 3.3. Synbiotics

In obesity therapy research, synbiotics, a combination of prebiotics and probiotics, have been investigated as a potential solution for gut microbiome dysfunction by employing complicated mixes of bacterial strains and varying prebiotic fiber concentrations [89]. Serving as a unique strategy for obesity prevention, combining omega-3 fatty acids with live probiotics has been shown to reduce hepatic steatosis and lipid buildup more significantly than probiotics alone [169,170]. Furthermore, diverse interventions, such as a combination of *Bacillus licheniformis* and xylo-oligosaccharides in obese rats and a combination of *Lactobacillus plantarum* PMO 08 with chia seeds in obese mice, show enhanced efficacy in improving body weight gain and lipid metabolism, as well as favorable changes in gut microbiota [171,172]. In addition, a combination of *Bifidobacterium lactis*, *Lactobacillus paracasei* DSM 4633, and oat β-glucan inhibited body weight gain and improved metabolic complications in obese mice [173]. This impact was achieved by restoring fecal levels of acetate, propionate, and butyrate while decreasing bile acid pools.

Formulations including *Clostridium butyricum* and corn bran reduce pathogen abundances, stimulate acetate-producing bacterial growth, and increase acetate and isovalerate synthesis [174]. Also, in an in vivo study, synbiotics containing *Lactobacillus paracasei* HII01 and xylo-oligosaccharides demonstrated the potential to prevent metabolic endotoxemia, decreasing the enrichment of Enterobacteriaceae and the Firmicutes to Bacteroidetes ratio in obese rats [127]. This intervention addressed the effects of an unhealthy diet that may promote the growth of LPS-producing bacteria, leading to LPS translocation caused by intestinal barrier compromise and subsequent metabolic disorders, insulin resistance, systemic inflammation, and immune responses [24].

However, while probiotic Bifidobacteria strains independently display anti-obesity effects, combining them with prebiotic galactooligosaccharides as a synbiotic does not result in synergistic benefits despite potential enhancements in the intestinal barrier function observed in obese adults [175]. This inconsistency could be attributed to the intensely competitive microenvironment, reminiscent of the gut microbiota, wherein substrates are concurrently accessible for both the indigenous microbiota and the introduced microbiota [89,150].

### 3.4. Postbiotics

Through complicated interactions with the immune system and food acquisition from the host, gut bacteria play a critical role in affecting host physiological processes by secreting low-molecular-weight metabolites that govern their development, growth, and propagation, as well as boosting the growth of beneficial species, allowing cell-to-cell contact, and protecting them from environmental challenges [56,176,177]. Some of these soluble mediators, known as postbiotics, can be produced by living bacteria or released following bacterial lysis and have the potential to benefit the host by altering cellular processes and metabolic functions [177]. Postbiotics come in a variety of forms. For example, the fermented infant formula (FIF) is made when infant formulas containing lactic acid-producing or other bacteria are fermented; it is typically devoid of viable bacteria, while paraprobiotics, also known as “ghost” probiotics, are non-viable or inactivated microbial cells that provide health benefits in sufficient quantities [177,178].

Other postbiotics include SCFA, peptides, enzymes, teichoic acids, and vitamins [177]. The gut microbiota produces SCFAs as metabolic byproducts when non-digestible carbohydrates—mainly acetate, propionate, and butyrate—are fermented [179]. The acetate/propionate ratio is important for de novo lipogenesis because, in contrast to acetate, butyrate, and propionate have been demonstrated to increase gut hormones and reduce food intake, stimulate intestinal gluconeogenesis, and cause the expression of genes linked to gluconeogenesis, leading to a decrease in body weight and fat deposition, while propionate inhibits hepatic lipogenesis by downregulating fatty acid synthase [89,180].

Exopolysaccharide from Lactobacillus plantarum L-14 and long-chain polyphosphate from Lactobacillus brevis both have therapeutic benefits in mice [24]. By stimulating the TLR2-AMPK signaling system, the former suppresses adipocyte development and regulates body weight and lipid profiles, whilst the latter accelerates intestinal epithelial wound healing and barrier function by activating the extracellular-regulated protein kinase (ERK) signaling pathway [89,90]. Additionally, postbiotics like muramyl dipeptide, derived from bacterial cell walls, alleviate obesity-induced insulin resistance by targeting nucleotide-binding oligomerization domain 2 (NOD2) and interferon regulatory factor 4 (IRF4), while interactions between muropeptide and NOD2 may improve insulin sensitization and alleviate inflammation [181,182].

## 4. Conclusions

Probiotics, prebiotics, synbiotics, and postbiotics all play a variety of roles that together have a wide range of effects on metabolic function. These constituents are essential for decreasing intestinal permeability by blocking LPSs and improving the function of the intestinal barrier by upregulating tight junction proteins and inhibiting proinflammatory pathways. As bioactive compounds, they modulate the gut microbiota and may aid in the complex reduction of chronic inflammation linked to obesity and related conditions. This multifaceted approach, which targets several aspects of gut health and immune functions, significantly reduces chronic inflammation through the regulation of the TLR family of proteins and inflammatory pathways contributing to obesity. Hence, by demonstrating the functional roles of probiotics, prebiotics, synbiotics, and postbiotics, future studies can seek to unravel the mechanism of action of probiotics, prebiotics, synbiotics, and postbiotics on TLRs in order to develop an effective therapeutic option for the management of obesity and related diseases.

## Figures and Tables

**Table 1:** Effect of probiotics, prebiotics, synbiotics, and postbiotics on inflammatory markers and intestinal barrier proteins in experimental animal models.

Target Diseases	Experimental Model	Bioactive Compounds	Doses	Effect on Inflammatory Markers and Intestinal Barrier Proteins	References
		Probiotics			
Diet-induced obesity and insulin resistance	Male C57BL/6J mice; 5-week-old	*Bifidobacterium lactis* LMG P-28149, and *Lactobacillus rhamnosus* LMG S-28148	5 × 10^8^ CFU	Decrease in epididymal adipose tissue expression levels of inflammatory cytokines *Tnfα, Il1a, Il6, and Il17*. Additionally, liver *Tnfα* and *Il6* were decreased while *Il10* expression was restored.	[120]
Diet-induced obesity	C57BL/6JRj male mice; 5-week-old	*Bifidobacterium longum* PI10 alone or a mixture of *Bifidobacterium animalis* subsp. lactis LA804 and *Lactobacillus gasseri* LA806	5 × 10^8^ CFU	Decrease in inflammatory-related genes *tnfα, mcp1,* and *cd68* in visceral adipose tissues; a significant decrease in jejunum *mcp1* gene expression.	[115]
Obesity and osteoporosis	Female C57BL/6 mice; 6 weeks old for GV-induced bacterial vaginosis and 11 weeks old for ovariectomy-induced osteoporosis	*Lactobacillus plantarum* NK3 and *Bifidobacterium longum* NK49 from kimchi	1 × 10^9^ CFU	Inhibition of NF-kB activation and TNF-α expression in the vagina, uterus, and colon; restoration of IL-10 expression in the vagina; and reduction in gut microbiota LPS production.	[14]
Type 2 diabetes	Female Wistar rats (120–160 g)	*Lactobacillus fermentum MCC2759* and *MCC2760*	10^9^ CFU	Downregulation of intestinal TNF-α, IL-1β, IL-6, and reduced expression of the TLR4 receptor while inducing the expression of IL-10, with a concomitant increase in the expression of tight junction proteins, ZO-1, GLP1, and endocannabinoid receptor CB2 in the intestine.	[121]
Hypertension	Wistar Kyoto rats; 5-week-old	*Bifidobacterium breve* *CECT7263* and *Lactobacillus fermentum* *CECT5716*	10^9^ CFU	Decreased plasma endotoxin (LPS) concentration; increased tissue repair of cytokine IL-18 expression. Together with SCFAs, the probiotics restored TLR4 mRNA levels and the aortic accumulation of Treg (except for acetate)	[122]
		Prebiotics			
Metabolic syndrome	Male *db*/*db* mice (C57BLKS/J-leprdb/leprdb); 5-week-old	oligofructose	0.6 g/day/mouse	Increased plasma levels of IL-10 and hypothalamic IL-1β mRNA expression; IL-6 expression was normalized by OFS; and blood–brain barrier integrity was improved due to normalized expression of ZO-1 and occludin.	[123]
Western-diet -induced obesity	Male C57BL/6N mice; 3–4 weeks old	oat and rye fiber	10% in diet;	Attenuation of liver mRNA expression levels TNF-α and TLR4; increased colonic mucin (Mucin 3) and tight junction protein (Occludin and Claudin 7) mRNA expression, suggesting improved gut barrier function	[124]
HF diet-induced obesity and diabetes	C57BL/6J mice; 8–10 weeks old	acorn and sago polysaccharides	5% in diet	Reduced expression of intestinal IL-6, TNF-α, chemokine (C-C motif) ligand 2 (Ccl2, and MCP1 with a corresponding increase in ZO-1 and Occludin.	[125]
Obesity and insulin resistance	Female C57BL/6NCrl mice; 15–16 weeks old	Isomaltodextrin	1%, 2.5%, and 5% introduced through drinking water	Decreased expression of plasma TNF-α and MCP-1; increased adiponectin levels and increased expression of mucin 2, mucin 4, and claudin 4.	[126]
		Synbiotics			
Obesity and insulin resistance	Male Wistar rats	Lactobacillus paracasei HII01 plus xylo-oligosaccharide (XOS)	10^8^ CFU; 10% XOS	Decrease in serum LPS levels, and the intestinal proinflammatory cytokine gene expression of IL-6 and IL-1β in the ileum and proximal colon	[127]
NAFLD	C57BL/6 mice; 4 weeks old	Lactobacillus paracasei N1115 (N1115) and fructooligosaccharides (FOS)	2.2 × 10^9^ CFU/mL (0.5 mL/day) and 4 g/kg/day	Decrease in serum and hepatic IL-1β and TNF-α, and serum LPS levels; decreased liver TLR4 and NF-κB mRNA expression; and increased intestinal protein expression of occludin-1 and claudin-1.	[128]
NAFLD	Male C57BL/6N mice	Bifidobacterium bifidum V, Lactobacillus plantarum X and Salvia miltiorrhiza polysaccharide	2 × 10^8^ CFU/mL; 1 × 10^8^ CFU/mL; 50 mg/kg/day	Reduced liver TNFα, IL-1β, and IL-6 mRNA expression alongside cecal LPSs.	[129]
		Postbiotics			
Obesity	C57BL/6J male mice; 4-week-old	Lactobacillus plantarum L-14 (KTCT13497BP) extract	500 mg/kg	Decreased expression of leptin, IL-6, TNF-α, and resistin with an increase in adiponectin and Arg1. Inhibition of adipogenesis via the activation of TLR2-AMPK pathway by exopolysaccharide	[130]
Type 2 diabetes	Male C57BL/6JUnib mice; 4–5 months old	Butyrate	5% *w*/*w*	Increase in claudin-1 levels within intestinal epithelia (jejunum, ileum, and colon)	[131]

**Table 2:** Select clinical trials on the impact of probiotics, prebiotics, synbiotics, and postbiotics on inflammatory markers and intestinal barrier proteins in overweight or obese subjects.

Participants/ Target Diseases	Bioactive Compounds	Doses	Effect on Inflammatory Markers and Intestinal Barrier Proteins	Research NCT	References
	Probiotics				
32 obese subjects aged 18–70 years with insulin resistance	Live or pasteurized *Akkermansia muciniphila*;	10^10^ CFU administered for 3 months	Decrease in LPSs, DPP-IV activity, chemokine GRO, MCP-1, γGT, AST, and sCD40 ligand, but no significant change in plasma GLP-1, ALT, or CRP levels; similarly, there was no change in visceral adiposity and BMI, but a slight decrease was seen in body weight	NCT02637115	[132]
51 patients with metabolic syndrome	*Bifidobacterium animalis* ssp. *lactis* ssp. nov. HN019	3.4 × 10^8^ CFU administered for 45 days	Decrease in TNF-α and IL-6		[133]
85 overweight or obese adults aged 25–65 years	*Lactobacillus paracasei (LCP-37),* *Lactobacillus acidophilus (NCFM),* *Bifidobacterium lactis (Bi-07),* and *Bifidobacterium lactis (Bi-04)*	1.25 × 10^9^ CFU administered for 8 weeks	Increase in hs-CRP while the IL-6 and ω6/ω3 ratio decreased; no change in IL-4, IL-8, IL12, 1L-10, IFN-γ, or TNF-α. Gut barrier protein levels for I-FABP and zonulin did not change	NCT04126330	[134]
69 obese or overweight adults aged 19–65 years	*Lactobacillus* *curvatus* *HY7601* *Lactobacillus plantarum KY1032*	5 × 10^9^ CFU administered for 12 weeks	Increase in hs-CRP, adiponectin, LDL-c and triglyceride levels		[135]
85 middle-aged overweight subjects	*Lactobacillus plantarum* *strain (LMT1-48)*	1 × 10^10^ CFU administered for 12 weeks	Increase in eGFR during 6th week but not during 12th week; decrease in the levels of ALT and AST during 6th and 12th weeks; decrease in total GIP during 12th week; no significant change in hs-CRP and GLP-1	NCT03759743	[136]
92 overweight or obese subjects aged 19 to 65 years;	*Limosilactobacillus fermentum MG4231 and MG4244*	2.5 × 10^9^ CFU administered for 12 weeks	Decrease in adiponectin and triglyceride levels; no significant change in hs-CRP, LDL-c or total cholesterol		[137]
81 obese subjects aged 20 to 65 years	*Lactobacillus plantarum K50 (LPK)*	2 × 10^9^ CFU administered twice daily for 12 weeks	Decrease in total cholesterol and triglyceride; no significant change in hs-CRP, LBP, resistin, and sCD14		[138]
101 obese youths aged 6–18 years with insulin resistance	*Bifidobacterium breve BR03* and *B632*	2 × 10^9^ CFU administered for 8 weeks	Decrease in ALT and IL-6 levels; increases in TNF-α and HDL-c	NCT03261466	[139]
44 obese subjects aged 20–60 years	*Bifidobacterium breve* CBT BR3, and *Lactobacillus* *plantarum* CBT LP3	1.5 × 10^10^ CFU administered for 12 weeks	TC/HDL increases significantly and slight increases in ALT and AST; no significant change in CRP		[140]
	Prebiotics				
26 overweight or obese adults aged 20–45 years	FOS (8.67 g) from Yacon flour (25 g)	Yacon flour consumed with a breakfast drink (350 mL) for 5 weeks	Significant increase in Nitric oxide level; No significant change in CRP, leukocyte, lymphocyte, platelet, or neutrophil levels		[141]
40 obese women aged 19–20 years	FOS (14 g) of Yacon syrup (14 g)	Yacon syrup administered for 2 days	No effect on GLP-1 levels		[142]
38 overweight or obese children aged 7–12 years	oligofructose	8 g of oligofructose-enriched inulin administered daily for 16 weeks	No significant changes in GIP, GLP-1, and PYY levels	NCT02125955	[143]
37 overweight or obese subjects aged 20–70 years	oligofructose	21 g of oligofructose administered for 12 weeks	Significant decrease in PAI-1 and LPSs; no changes in IL-6, TNF-α, MCP-1, adiponectin or resistin	NCT00522353	[144]
48 obese subjects ≥30 years	Dietary fiber	16 g of dietary (study beans) fiber administered for 6 weeks	Increase in FGF-19; decrease in IL10rα, TRANCE, CD8A, PD-L1, CXCL1, and uPA	NCT02843425	[145]
45 obese and major depressive disorder subjects aged 20–50 years	inulin	10 g of Frutafit (inulin/oligofructose) daily for 8 weeks	No significant effect on endotoxemia LPSs, gut barrier protein Zonulin, BDNF, or the inflammatory markers IL-10, TNF-α, MCP-1, TLR-4, and hs-CRP levels		[146]
24 subjects aged 18–65 years with an obesity-related metabolic disorder	Inulin	16 g of native inulin (obtained from chicory root, Belgium) daily for 3 months	Decrease in calprotectin but no change in fecal zonulin	NCT03852069	[147]
14 overweight or obese male adults aged 20–50 years	Inulin	24 g of inulin administered over a 2-day investigation	No significant change in plasma GLP-1 and PYY	NCT02009670	[148]
	Synbiotics				
29 overweight or obese subjects aged 20–60 years	*Bifidobacterium lactis* HN019; *Lactobacillus acidophilus* NCFM; and polydextrose	1 × 10^10^ CFU of probiotics and 1.7 g of polydextrose administered for eight weeks	No significant change in CRP or lipid profile	NCT05459909	[149]
76 overweight or obese subjects aged 50–70 years	*Bifidobacterium breve,* *Bifidobacterium longum,* *Lactobacillus acidophilus,* *Lactobacillus bulgaricus,* *Lactobacillus casei,* *Lactobacillus rhamnosus,* and *Streptococcus thermophiles;* *FOS*	10^9^ CFU of probiotics and 35 mg of FOS administered daily for 8 weeks	Increased adiponectin and decreased TNF-α, hs-CRP levels		[150]
86 overweight or obese subjects aged 30–80 years	*Lacticaseibacillus paracasei YIT 9029;* *Bifidobacterium breve YIT 12272;* and GOS	3 × 10^8^ CFU live probiotics and 7.5 g of GOS daily for 24 weeks	No significant effect on IL-6, LBP, or hs-CRP		[151]
56 overweight or obese subjects with a mean age of 40.8 ± 14 years	*Bifidobacterium lactis* W51 (NIZO 3680), *Bifidobacterium lactis* W52 (NIZO 3882), *Lactobacillus acidophilus* W22 (NIZO 3674), *Lactobacillus paracasei* W20 (NIZO 3672), *Lactobacillus plantarum* W21 (NIZO 3673), *Lactobacillus salivarius* W24 (NIZO 3675), and *Lactococcus lactis* W19 (NIZO 3671); FOS *and* Inulin	0.9–2.8 (×10^8^) CFU probiotics plus 9.6 mg of FOS and 110.4 mg of inulin administered for 12 weeks	Decrease in fecal zonulin level		[152]
26 patients aged >18 years with diabesity	*B. bifidum* W23, *B. lactis* W51, *B. lactis* W52, *L. acidophilus* W37, *L. casei* W56, *L. brevis* W63, *L. salivarius* W24, *Lc. lactis* W58 and *Lc. lactis* W19; FOS, GOS, and konjac glucomannan P13 (E425)	1.5 × 10^10^ CFU probiotics and 8 g of active prebiotics administered for 6 months	Reduction in serum zonulin levels after 3 months but not 6 months; no significant changes to LPS, LBP, and sCD14 levels	NCT02469558	[153]
41 adults aged 30–65 years with obesity or hyperglycemia	INN pasta containing *Bacillus coagulans GBI-30 6086* and barley β-glucans; 7 log CFU/g (10 million CFU/g)	1 serving of INN pasta taken for 12 weeks	Increase in plasma IL-6; decrease in plasma hs-CRP	NCT02236533	[154]
94 adults aged 18–65 years with obesity	*Bifidobacterium* *adolescentis* IVS-1 and *Bifidobacterium animalis* subsp. *lactis* BB-12; GOS	1 × 10^9^ CFU for each probiotic and 6.9 g of GOS administered daily for 3 weeks	No noticeable change in endotoxemia markers of LPS and LBP; reduced ratio of post-aspirin sucralose to lactulose	NCT02355210	[155]
	Postbiotics				
49 overweight adults aged 21–65 years	Propionate	10 g of inulin-propionate ester administered daily for 24 weeks	Increase in postprandial plasma PYY and GLP-1	NCT00750438	[156]
6 overweight or obese adult men	Acetate	100 or 180 mmol/L colonic acetate	Increased fasting PYY; slight decrease in TNF-α		[157]
12 overweight or obese adult men aged 20–40 years	Acetate, butyrate, and propionate	8–24 mmol (20–60%) of sodium acetate, sodium propionate, and sodium butyrate in 200 mL of sterile water administered for 4 days	Increase in PYY, and postprandial GLP-1; no significant change in ANGPTL4, TNF-α, IL-6 and IL-8;		[158]
48 children aged 5–17 years with pediatric obesity	Butyrate	20 mg/kg of body weight of sodium butyrate administered daily for 6 months	Reduction in microRNA-221, and IL-6	NCT04620057	[159]

## Abbreviations

DPP-IV: dipeptidyl peptidase-IV, CRP: C-reactive protein, GLP-1: glucagon-like peptide-1, GRO: growth-regulated oncogene, MCP-1: monocyte chemoattractant protein-1, γGT: gamma-glutamyl transferase (γGT), AST: aspartate aminotransferase, ALT: alanine-aminotransferase, I-FABP: intestinal fatty acid-binding protein, eGFR: estimated glomerular filtration rate, GIP: gastric inhibitory polypeptide: PYY: peptide YY, IL10rα: interleukin 10 receptor alpha, FGF-19: fibroblast growth factor, TRANCE: tumor necrosis factor-related activation-induced cytokine, CD8A: T-cell surface glycoprotein CD8 alpha chain, PD-L1: programmed cell death 1 ligand 1, CXCL1: growth-regulated alpha protein C-X-C motif chemokine 1, uPA: urokinase plasminogen activator, BDNF: brain-derived neurotrophic factor, GOS: galacto-oligosaccharides, LBP: lipopolysaccharide-binding protein, ANGPTL4: angiopoietin-like protein 4, TLR: toll-like receptor, and NF-κB: nuclear factor kappa B.
